# Effect of Systemic Immune-Inflammation Index on Prognosis in Non-Functional Mitral Regurgitation Patients Undergoing Isolated Mitral Valve Replacement

**DOI:** 10.21470/1678-9741-2023-0362

**Published:** 2025-06-11

**Authors:** Busra Temel Yuksel, Mehmet Isık, Omer Tanyeli, Serkan Yıldırım, Niyazi GOrmus

**Affiliations:** 1 Department of Cardiovascular Surgery, Şırnak State Hospital, Şırnak, Turkey; 2 Department of Cardiovascular Surgery, Necmettin Erbakan University Faculty of Medicine Hospital, Konya, Turkey

**Keywords:** Lymphocytes, Blood Platelets, Neutrophils, Mitral Valve Insufficiency, Mortality, Prognosis

## Abstract

**Objective:**

To investigate the effect of preoperative and postoperative systemic immune-inflammation index (SII) values on early prognosis in patients with nonfunctional mitral regurgitation etiology undergoing isolated mitral valve replacement (MVR).

**Methods:**

A total of 176 patients with isolated MVR performed from 2015 to 2021 were retrospectively investigated. The platelet, lymphocyte, and neutrophil counts were measured, and SII, neutrophil/lymphocyte ratio (NLR), and platelet/lymphocyte ratio (PLR) values were calculated preoperatively and on the first and fourth days postoperatively. The correlations with postoperative 30-day early-term prognosis and mortality were investigated.

**Results:**

Mean age of the patients was 55.4 years, 69.9% were female, and 30.1% were male. At 30-day follow-up, 9% (n = 16) of patients died. There were significant positive correlations observed between age (P < 0.001), preoperative NLR (P = 0.003), preoperative SII (P = 0.02), and postoperative fourth day NLR (P < 0.001) values with 30-day mortality. Receiver operating characteristic analysis identified that age + preoperative SII (P < 0.001), age + preoperative NLR (P < 0.001), and age + postoperative fourth day NLR (P = 0.001) combinations were significant predictive factors for 30-day mortality. There was a significant positive correlation between postoperative fourth day SII value with intensive care unit (ICU) admission duration (P < 0.001, Ρ = 0.308).

**Conclusion:**

For non-functional, isolated MVR patients, preoperative and postoperative fourth day SII and NLR values were found to provide an idea about 30-day prognosis. Additionally, patients with high postoperative fourth day SII values were observed to have longer ICU stays.

## INTRODUCTION

**Table:** 

Abbreviations, Acronyms & Symbols				
AF	= Atrial fibrillation		MR	= Mitral regurgitation
AUC	= Area under the curve		MVR	= Mitral valve replacement
BMI	= Body mass index		NLR	= Neutrophil/lymphocyte ratio
CI	= Confidence interval		PLR	= Platelet/lymphocyte ratio
CPB	= Cardiopulmonary bypass		ROC	= Receiver operating characteristic
CRP	= C-reactive protein		SD	= Standard deviation
ICU	= Intensive care unit		SII	= Systemic immune-inflammation index
LL	= Log likelihood			

Mitral regurgitation (MR) increases with age, and prevalence rises to nearly 9% in the population over 75 years of age^[1]^. Mitral valve replacement (MVR) still has an important place for surgical treatment of MR patients unsuitable for repair. Easily applied markers are being researched to gain an idea about prognosis of patients after MVR.

There are several markers that indicate the presence of systemic inflammation. Hemogram is the most frequently used parameter because it is cheap and gives quick and easy results. Neutrophil/lymphocyte ratio (NLR), among leukocyte subgroups, platelet count, and C-reactive protein (CRP) are indicators of immune inflammatory activity providing significant results in clinical practice^[2^-^4]^. In recent years, a new marker called systemic immune-inflammation index (SII) (platelet count × NLR) has been developed. As this formula includes three cell types, it was proposed to provide valuable information about inflammation[2].

SII and/or NLR were previously shown to be beneficial markers to predict poor clinical outcomes in cancer patients and inflammatory disease cases^[5]^. Later, studies were performed for cardiovascular diseases and they were found to be good markers for in-hospital negative clinical outcomes, carotid artery disease, and postoperative atrial fibrillation (AF)^[6^-^9]^.

In this study, the aim was to investigate the effect of SII values measured preoperatively and postoperatively on early-term prognosis in patients with non-functional MR etiology undergoing MVR.

## METHODS

The files of 450 patients with isolated MVR performed due to primary MR from January 2015 to December 2021 in our clinic were investigated. The study included 176 patients with nonfunctional MR etiology who underwent elective surgery with sternotomy. Exclusion criteria were emergency cases, infective endocarditis, mitral repair, left atrial mass, reoperations, ischemic MR etiology, patients under 18 years, MVR with thoracotomy, pregnant or lactating women, those with autoimmune disease, and patients undergoing combined cardiac surgical procedures.

Information about patients was obtained from the hospital software system, medical records, doctor observation notes, and the national health system database. Patients provided written informed consent for the surgical procedure, and our retrospective study received protocol permission from the Necmettin Erbakan University Faculty of Medicine ethics committee (2022/3653). The study assessed postoperative 30-day early-term outcomes.

The patients were grouped according to age (18-49, 50-65, and > 65 years), type of valve used (mechanical, biological), and diagnosis (degenerative, rheumatological).

Preoperative and postoperative blood parameters (hematocrit, neutrophil, lymphocyte count, platelet, CRP), preoperative and postoperative 30-day echocardiogram findings (ejection fraction, etiology), dimension of valve used, total pump duration, aortic cross-clamping duration, presence of AF, intensive care unit (ICU) and in-hospital stays, 30-day prognosis, and mortality data were recorded for patients.

Each patient had SII, NLR, and platelet/lymphocyte ratio (PLR) values calculated using the platelet, lymphocyte, and neutrophil counts measured preoperatively, and on the first and fourth days postoperatively. The correlations of the results with postoperative prognosis and mortality were investigated.

The following formula was used to calculate the markers:

SII (× 10^9^/L) = platelet count (K/µL) (P) * neutrophil count (K/µL) (N)/lymphocyte count (K/µL) (L) (SII=P*N/L)

Where L is lymphocyte, N is neutrophil, and P is platelet.

### Surgical Procedure

Following general anesthesia, median sternotomy was performed. With aorta and selective bicaval venous cannulation, cardiopulmonary bypass (CPB) was initiated. During cross-clamping duration, body temperature was lowered to mean 32° C, and cold blood cardioplegia was repeated every 20 minutes. With transseptal right atriotomy incision, the mitral valve was reached. The posterior leaflet was protected as much as possible. With the single pledged suture technique, mechanical or bioprosthetic valves, as appropriate for the patient, were inserted in the annulus. The interatrial septum was closed with 3/0 monofilament non-absorbable sutures. For CPB, roller pump and membrane oxygenator were used. The surgical team was the same for all cases.

### Statistical Analysis

Analyses were completed with IBM Corp. Released 2012, IBM SPSS Statistics for Windows, version 21.0, Armonk, NY: IBM Corp. software. In our study, categoric and quantitative data were defined, and numerical parameters are given as median or mean ± standard deviation while categoric parameters are presented as percentage and frequency. Fit of numerical data to normal distribution was tested with skewness-kurtosis, Kolmogorov-Smirnov test, and histogram analyses. Based on the features of parameter distribution, comparison of two independent groups used the independent sample *t*-test or Mann-Whitney U test. Based on parameter distribution, comparison of three or more dependent groups used the Friedman test or repeated analysis of variance test. Binary logistic regression analysis and models were created for effect level analysis and diagnostic data, and the regression fit of the models was confirmed with the Box-Tidwell test. Compatibility of parameters for logistic regression analysis was checked with the Hosmer-Lemeshow test. Effect level and predictive ability of parameters for 30-day mortality were examined in detail with receiver operating characteristic (ROC) analysis, and diagnostic data were researched. Our study was performed with 5% type-I error rate, and *P* < 0.05 was accepted as the limit of significance.

## RESULTS

Of patients, 123 were women (69.9%) and 53 were men (30.1%) with mean age of 55.4 years (21-85) and mean body mass index (BMI) 27.5 kg/m2 (14.5-47.5). The median value for total pump duration was 89 minutes (56-166), with median value for aortic cross-clamping duration of 58 minutes (36-127). Median stay in intensive care was two days (1-26), and median total hospitalization stay was eight days (1-42). When etiology is investigated, 58% were in the rheumatic heart valve disease group and 42% were in the degenerative diagnostic group. Demographic data are summarized in Table 1.

**Table 1:** Baseline and clinical characteristics (n = 176).

		Rheumatological disease n = 102	Degenerative disease n = 74	Bioprosthesis n = 25	Mechanical prosthesis n = 151
Age (years)	19-49 (n = 54)	54	-	-	54
	50-65 (n = 82)	48	34	-	82
	> 65 (n = 40)	-	40	25	15
Sex	Male (n = 53)	18	35	11	42
	Female (n = 123)	84	39	14	109
Diabetes mellitus	(n = 29)	8	21	6	23
Hypertension	(n = 104)	46	58	18	86
Kidney disease	(n = 22)	12	10	10	12
Atrial fibrillation	(n = 99)	56	43	14	85
Asthma	(n = 31)	18	13	7	24
Dyslipidemia	(n = 34)	9	25	15	19
Cerebrovascular accident	(n = 11)	4	7	6	5
Cigarette smoking	(n = 51)	33	18	7	44
Preoperative ejection fraction	(%)	^*^60 (25-70)	^*^60 (28-65)	^*^60 (30-65)	^*^60 (25-70)

^*^Median value

**Fig. 1:**
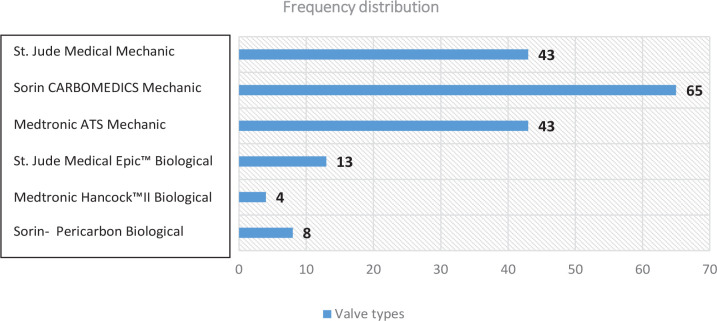
Frequency distribution of valve types.

According to the frequency of valve types used, the order was Sorin CARBOMEDICS™ mechanical (n = 65, 36.9%), St. Jude Medical mechanical (n = 43, 24.4%), and Medtronic ATS mechanical (n = 43, 24.4%), St. Jude Medical Epic™ bioprosthetic (n = 13, 7.4%), Sorin-Pericarbon bioprosthetic (n = 8, 4.5%), and Medtronic Hancock™ II bioprosthetic (n = 4, 2.3%) (Figure 1). Of the valves used, 85.8% (n = 151) were mechanical and 14.2% (n = 25) were bioprosthetic valves. The median value for valve sizes was 29 (24-35). Distribution of mortality was 28.0% in the bioprosthetic valve group (n = 7) and 6.0% in the mechanical valve group (n = 9) (*P* = 0.002). Among the mortality group, 25% were men and 75% were women.

The progression of laboratory parameters (CRP, platelet, neutrophil, lymphocyte) and some proportional values (NLR, PLR, SII) were analyzed preoperatively and on the postoperative first and fourth days, and statistical correlations were identified. Significant statistical correlations were observed between groups for all parameters (Table 2).

**Table 2:** Analysis of laboratory outcomes in three different time periods (preoperative, postoperative first day, and postoperative fourth day).

		Preoperative	Postoperative 1^st^ day	Postoperative 4^th^ day	*P*-value
Variables	Unit	Median (min-max) or mean ±SD			
CRP	mg/L	4.0 (0.1-142.0)	41.0 (7.0-146.0)	136.0 (19.0-412.0)	< 0.001
Plateletsǂ	(K/µL)	271.8 ± 81.5	205.9 ± 61.8	171.8 ± 58.1	< 0.001^*^
Neutrophil	(K/µL)	4.7 (1.2-19.6)	12.3 (3.1-30.0)	7.5 (1.9-24.3)	< 0.001
Lymphocyte	(K/µL)	2.1 (0.62.0-4.0)	0.7 (0.19-2.2)	1.6 (0.10-4.6)	< 0.001
NLR		2.33 (0.57-11.70)	17.93 (3.95-64.21)	4.94 (1.67-58.65)	< 0.001
PLR		128.25 (46.67-1238.0)	286.48 (49.28-1035.0)	107.05 (40.56-19.23)	< 0.001
SII	× 10^9^/L	592.11(125.14-5226.67)	3772 (433.7-16353.0)	801.9 (163.61-5952.0)	< 0.001

ǂThe results are based on mean values

* Mauchly's sphericity test H₀ hypothesis was rejected and Greenhouse-Geiser correction was made; F (1.709) = 210.76 (*P* < 0.001)

CRP=C-reactive protein; NLR=neutrophil/lymphocyte ratio; PLR=platelet/lymphocyte ratio; SD=standard deviation; SII=systemic immune-inflammation index

The correlations for progression of SII values preoperatively and on the postoperative first and fourth days were separately analyzed for age, sex, valve group, diagnosis group, and 30-day prognosis group categories. Investigations observed significant statistical relationships between groups for all parameters (Table 3). For all groups, the SII values were higher on the postoperative first day, preoperative SII values were the lowest, and this represented statistical significance (Table 3).

**Table 3:** Analysis of preoperative, postoperative first day, and postoperative fourth day SII outcomes of patients according to age, sex, valve group, diagnosis group, and 30-day mortality status.

Parameter		Group	Preoperative	Postoperative 1^st^ day	Postoperative 4^th^ day	*P*-value
			Median (min-max)			
SII (× 10^9^/L)	Sex	Male	584.57 (192.50-5226.67)	3436.36 (1030.86-14150.0)	830.8 (303.73-5952.0)	< 0.001
		Female	594.12 (125.14-3478.22)	3777.66 (433.71-16353.0)	764.15 (163.61-5200.0)	< 0.001
	Age	18-49 years	598.40 (172.06-3460.44)	3689.68 (1003.30-9844.8)	695.31 (263.33-3915.0)	< 0.001
		50-65 years	568.30 (166.96-5226.6)	3659.51 (433.71-16353.0)	832.82 (203.67-5200.)	< 0.001
		> 65 years	615.0 (125.14-2691.0)	3917.33 (815.79-11662.8)	1048.0 (163.61-5952.0)	< 0.001
	Valve group	Bioprosthetic	644.0 (125.14-2691.0)	4094.8 (815.79-11662.8)	1053.29 (163.61-5952.0)	< 0.001
		Mechanical	590.90 (166.96-5226.67)	3596.02 (433.71-16353.0)	752.56 (203.67-5200.0)	< 0.001
	Diagnostic group	Rheumatological	601.25 (166.96-5226.67)	3576.05 (730.91-16353.0)	791.43 (203.67-5200.0)	< 0.001
		Degenerative	776.01 (125.14-3478.22)	3990.0 (433.71-11662.86)	819.69 (163.61-5952.0)	< 0.001
	Prognosis^*^	Survival	580.16 (125.14-5226.67)	3795.98 (734.40-16353.0)	780.0 (163.61-5200.0)	< 0.001
		Mortality	785.13 (362.40-3478.22)	2451.40 (433.71-11662.86)	1263.27 (499.50-5952.0)	0.004

* Results are based on 30-day mortality (mortality/survival)

The presence of correlations between preoperative SII, postoperative first day SII, and postoperative fourth day SII with ICU admission and ward admission was investigated. According to Spearman two-tailed correlation analysis, there was a statistically significant, non-linear positive correlation identified between the postoperative fourth day SII value with ICU admission (*P* < 0.001, *Ρ* = 0.308). No significant correlations were identified for the other parameters.

The correlation between operation data with hospitalization, valve groups (mechanical/bioprosthetic), and diagnosis groups (degenerative/rheumatic) was analyzed. There was no statistically significant difference observed between the mechanical and bioprosthetic valve groups for operation data and hospitalization. In the degenerative diagnosis group, only median total pump duration (92 [61-166] minutes) was found to be higher compared to the rheumatic heart valve disease group (87 [56-146] minutes), and this was statistically significant (*P* = 0.03). There was no significant correlation identified between operation duration and mortality (*P* > 0.7).

The correlation between preoperative laboratory parameters and some proportional values (NLR, PLR, SII) with valve groups (bioprosthetic/mechanical) and diagnosis groups (rheumatological/degeneration) was researched. No significant difference was observed between the groups for preoperative SII values (*P* > 0.1).

The correlations between postoperative first day laboratory parameters and some proportional values (NLR, PLR, SII) with valve groups (bioprosthetic/mechanical) and diagnosis groups (rheumatological/degeneration) were researched. Significant differences were not observed for the valve groups (Table 4). The postoperative first day NLR value (*P* = 0.04) was higher in the degenerative valve disease group, while lymphocyte values (*P* = 0.02) were higher in the rheumatological valve disease group. There was no significant difference observed between rheumatological and degenerative groups in terms of postoperative first day SII values (*P* = 0.47) (Table 4).

**Table 4:** Postoperative first day outcomes according to valve used and diagnosis groups.

		Bioprosthetic (n = 25)	Mechanical (n = 151)	*P*-value
Variables	Unit	Median (min-max) or mean ± SD		
CRP	mg/L	29.0 (10.0-97.0)	43.0 (7.0-146.0)	0.29
Plateletǂ	(K/µL)	190.37 ± 79.87	208.40 ± 58.48	0.29
Neutrophil	(K/µL)	12.2 (3.1-26.0)	12.3 (5.4-30.0)	0.88
Lymphocyte	(K/µL)	0.63 (0.19-1.4)	0.71 (0.27-2.2)	0.17
NLR		20.73 (6.55-64.21)	17.11 (3.95-56.29)	0.19
PLR		280.63 (126.43-757.89)	286.48 (49.28-1035.0)	0.96
SII	× 10^9^/L	4094.87 (815.79-11662.86)	3596.02 (433.71-16353.0)	0.77
		Rheumatological valve diseases (n = 102)	Degenerative valve diseases (n = 74)	*P*-value
Variables	Unit	Median (min-max) or mean ± SD		
CRP	mg/L	44.5 (7.0-146.0)	31.0 (9.0-116.0)	0.07
Plateletǂ	(K/µL)	213.83 ± 56.11	194.89 ± 68.02	0.053
Neutrophil	(K/µL)	12.8 (5.4-30.0)	11.4 (3.1-26.6)	0.54
Lymphocyte	(K/µL)	0.74 (0.27-2.2)	0.64 (0.19-1.5)	0.02
NLR		16.50 (3.95-53.33)	20.14 (5.53-64.21)	0.04
PLR		285.74 (54.55-1035.0)	287.27 (49.28-757.89)	0.58
SII	× 10^9^/L	3576.0 (730.9-16353.0)	3990.0 (433.71-11662.8)	0.47

CRP=C-reactive protein; NLR=neutrophil/lymphocyte ratio; PLR=platelet/lymphocyte ratio; SD=standard deviation; SII=systemic immune-inflammation index

ǂThe results are based on mean values

The correlations between postoperative fourth day laboratory parameters and some proportional values (NLR, PLR, SII) with valve groups and diagnosis groups were researched. The investigation found higher postoperative fourth day NLR (*P* < 0.001) and PLR (*P* = 0.006) values in the bioprosthetic group. However, postoperative fourth day platelet (*P* = 0.02) and lymphocyte values (*P* < 0.001) were higher in the mechanical valve group. There were no significant statistical differences observed between the groups for the other parameters (Table 5). Investigations of the diagnostic groups did not observe a significant difference in terms of postoperative fourth day SII values (*P* = 0.67) between the rheumatological and degenerative groups. Patients with degenerative valve disease appeared to have higher postoperative fourth day NLR (*P* = 0.01), and this represented a significant difference. However, the postoperative fourth day platelet (*P* = 0.003) and lymphocyte (*P* < 0.001) values were higher in the group with rheumatic heart valve disease, and this was significant. Significant differences were not observed between the groups for the other parameters (Table 5).

**Table 5:** Postoperative fourth day outcomes according to valve used and diagnosis groups.

		Bioprosthetic (n = 25)	Mechanical (n = 151)	*P*-value
Variables	Unit	Median (min-max) or mean ± SD		
CRP	mg/L	152.5 (38.0-412.0)	135.0 (19.0-360.0)	0.71
Plateletǂ	(K/µL)	145.36 ± 64.24	175.85 ± 56.38	0.02
Neutrophil	(K/µL)	7.2 (1.9-18.6)	7.5 (3.2-24.3)	0.76
Lymphocyte	(K/µL)	1.0 (0.10-1.9)	1.7 (0.2-4.6)	< 0.001
NLR		7.29 (4.26-58.65)	4.58 (1.67-41.35)	< 0.001
PLR		156.12 (54.21-519.23)	105.23 (40.56-400.0)	0.006
SII	× 10^9^/L	1053.2 (163.61-5952.0)	752.8 (203.67-5200.0)	0.053
		Rheumatological valve diseases (n = 102)	Degenerative valve diseases (n = 74)	*P*-value
Variables	Unit	Median (min-max) or mean ± SD		
CRP	mg/L	140.0 (19.0-360.0)	130.0 (25.0-412.0)	0.32
Plateletǂ	(K/µL)	178.0 ± 53.20	150.50 ± 61.57	0.003
Neutrophil	(K/µL)	7.9 (3.2-20.8)	7.0 (1.9-24.3)	0.21
Lymphocyte	(K/µL)	1.8 (0.37-4.6)	1.4 (0.1-3.6)	< 0.001
NLR		4.57 (1.67-41.35)	6.26 (1.67-58.65)	0.01
PLR		101.76 (52.22-400.0)	116.90 (40.56-519.23)	0.07
SII	× 10^9^/L	791.43 (203.67-5200.0)	819.69 (163.61-5952.0)	0.67

ǂThe results are based on mean values

CRP=C-reactive protein; NLR=neutrophil/lymphocyte ratio; PLR=platelet/lymphocyte ratio; SD=standard deviation; SII=systemic immune-inflammation index

In the 30-day prognosis (death/survival) analysis, it was observed that 9% (n = 16) of the patients died; 75% of the deceased patients were women and 25% were men. On the other hand, 30.6% of the surviving group were men and 69.4% were women. The existence of a relationship between the valve groups and diagnosis groups used and the 30-day prognosis (death/survival) was examined. It was observed that 28.0% (n = 7) of the patients using bioprosthetic valves died, while 6.0% (n = 9) of the patients using mechanical valves died, and the analysis was statistically significant (*P* = 0.002). In the analysis of diagnostic groups, it was seen that 3.9% (n = 4) of the rheumatological valve group died and 16.2% (n = 12) of the degenerative valve patients died, and this difference was statistically significant (*P* = 0.005). The mean age was 52.70 ± 11.20 years in the mechanical valve group and 72.08 ± 6.91years in the bioprosthetic group. In the examination performed in terms of hospitalization time, no significant difference was found between the biological and mechanical valve groups.

The correlations between preoperative, postoperative first day, and postoperative fourth day parameters and some proportional values (NLR, PLR, SII) with 30-day prognosis was researched. The investigation observed that preoperative CRP (*P* = 0.003), NLR (*P* = 0.003), and SII (*P* = 0.02) values were higher in the mortality group. However, neutrophil values (*P* = 0.01) were lower in the mortality group. Parameters examined on the postoperative first day did not have significant statistical differences between the groups (Table 6). Of the postoperative fourth day parameters, NLR (*P* < 0.001) was higher in the mortality group, and this represented a statistically significant difference. However, platelet (*P* = 0.001) and lymphocyte (*P* = 0.002) values were lower in the mortality group on the postoperative fourth day, and this was statistically significant. No significant differences were observed between the groups for the other parameters (Table 6).

**Table 6:** Preoperative, postoperative first, and postoperative fourth day results according to 30-day survival status.

Preoperative		Survival (n = 160)	Mortality (n = 16)	*P*-value
Variables	Unit	Median (min-max) or mean ± SD		
CRP	mg/L	3.50 (0.1-101.0)	20.0 (1.0-142.0)	0.003
Plateletǂ	(K/µL)	270.35 ± 79.95	291.25 ± 112.52	0.34
Neutrophil	(K/µL)	4.6 (1.2-19.6)	6.0 (3.2-15.7)	0.01
Lymphocyte	(K/µL)	2.1 (0.62-4.0)	1.8 (1.0-3.6)	0.41
NLR		2.23 (0.57-11.70)	2.93 (2.00-6.22)	0.003
PLR		126.16 (48.13-1238.0)	167.33 (46.67-310.56)	0.17
SII	× 10^9^/L	580.16 (125.14-5226.67)	789.06 (387.33-3478.22)	0.02
Postoperative 1^st^ day		Survival (n = 160)	Mortality (n = 16)	*P*-value
Variables	Unit	Median (min-max) or mean ± SD		
CRP	mg/L	42.5 (7.0-116.0)	30.0 (10.0-146.0)	0.44
Plateletǂ	(K/µL)	208.26 ± 60.09	181.00 ± 76.56	0.10
Neutrophil	(K/µL)	12.2 (3.1-30.0)	13.4 (8.8-26.0)	0.30
Lymphocyte	(K/µL)	0.70 (0.19-2.0)	0.70 (0.40-2.2)	0.34
NLR		17.87 (3.95-64.21)	20.60 (6.28-37.14)	0.84
PLR		288.95 (115.22-1035.0)	212.50 (49.28-650.0)	0.09
SII	× 10^9^/L	3795.98 (734.40-16353.0)	2451.40 (433.71-11662.8)	0.35
Postoperative 4^th^ day		Survival (n = 160)	Mortality (n = 16)	*P*-value
Variables	Unit	Median (min-max) or mean ± SD		
CRP	mg/L	137.0 (19.0-412.0)	135.0 (25.0-256.0)	0.80
Plateletǂ	(K/µL)	175.26 ± 56.93	111.89 ± 48.74	0.001
Neutrophil	(K/µL)	7.4 (1.9-24.3)	10.8 (3.7-19.8)	0.10
Lymphocyte	(K/µL)	1.6 (0.1-4.6)	0.9 (0.2-1.8)	0.002
NLR		4.75 (1.67-58.65)	11.66 (6.55-46.50)	< 0.001
PLR		105.7 (42.20-519.23)	151.0 (40.56-320.0)	0.15
SII	× 10^9^/L	780.0 (163.6-5200.0)	1263.2 (499.5-5952.0)	0.11

ǂThe results are based on mean values

CRP=C-reactive protein; NLR=neutrophil/lymphocyte ratio; PLR=platelet/lymphocyte ratio; SD=standard deviation; SII=systemic immune-inflammation index

Logistic regression analysis was performed for the correlations between age, sex, BMI, and SII values at the three different time points with 30-day mortality. The investigation observed significant positive correlations between age (*P* < 0.001), preoperative SII values (*P* = 0.049), and postoperative fourth day SII values (*P* = 0.04) with mortality. The other parameters were not observed to have significant effect on mortality (Table 7).

**Table 7:** Logistic regression analysis and effect levels of some variables that can be used in the prediction of 30-day mortality at first admission.

Mortality (*Exitus*)						
Variables	B	-2LL	R2Nagelkerke	*P*-value	Exp(B)	95% CI
Sex	-0.281	107.006	0.003	0.75	0.75	0.232-2.459
Age (years)	0.108	89.666	0.208	< 0.001	1.115	1.051-1.182
BMI (kg/m^2^)	-0.051	106.104	0.012	0.34	0.950	0.854-1.057
Preoperative SII^*^ (× 10^9^/L)	0.001	103.817	0.042	0.049	10.005	1.0000-1.001
Postoperative 1^st^ day SII^*^ (× 10^9^/L)	-0.00007	102.010	0.005	0.56	0.999	0.999-1.000
Postoperative 4^th^ day SII^*^ (× 10^9^/L)	0.001	66.815	0.060	0.04	10.005	1.0000-1.001

Reference category: survival group

* It was seen as theoretically meaningful, but with poor clinical applicability

BMI=body mass index; CI=confidence interval; LL=Log likelihood; SII=systemic immune-inflammation index

**Fig. 2:**
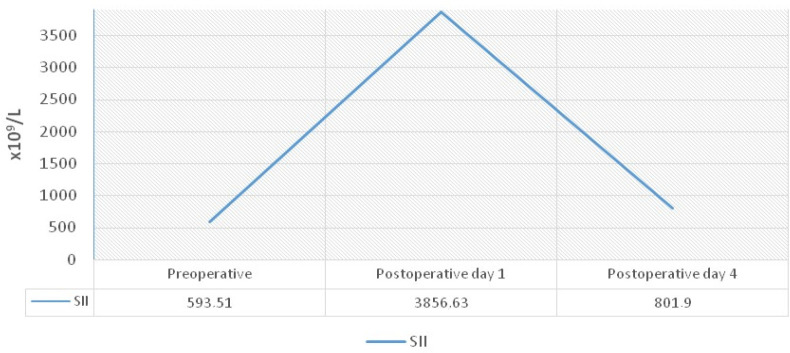
The course of systemic immune-inflammation index (SII) values of patients in different time periods.

The SII values at the three different time points underwent ROC analysis for predicting 30-day mortality. Preoperative SII was observed to have predictive features (592.1 cut-off value, sensitivity 81.3%, specificity 52.5%, area under the curve [AUC] 0.676, *P* = 0.02). The course of SII in different periods is shown in Figure 2.

**Fig. 3:**
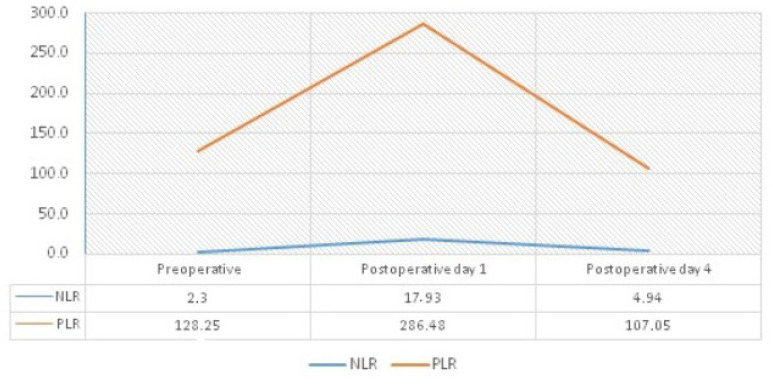
The course of neutrophil/lymphocyte ratio (NLR) and platelet/lymphocyte ratio (PLR) values of patients in different time periods.

The NLR and PLR values at three different time points were analyzed with ROC for prediction of 30-day mortality. The preoperative NLR value was found to have predictive features (2.631 cut-off values, sensitivity 62.5%, specificity 64.4%, AUC 0.722, *P* = 0.003). The postoperative fourth day NLR value was also found to have predictive features (8.125 cut-off, sensitivity 77.8%, specificity 78.1%, AUC 0.883, *P* < 0.001). The other parameters were not observed to have significant predictive features. The course of NLR and PLR in different periods is shown in Figure 3.

**Fig. 4:**
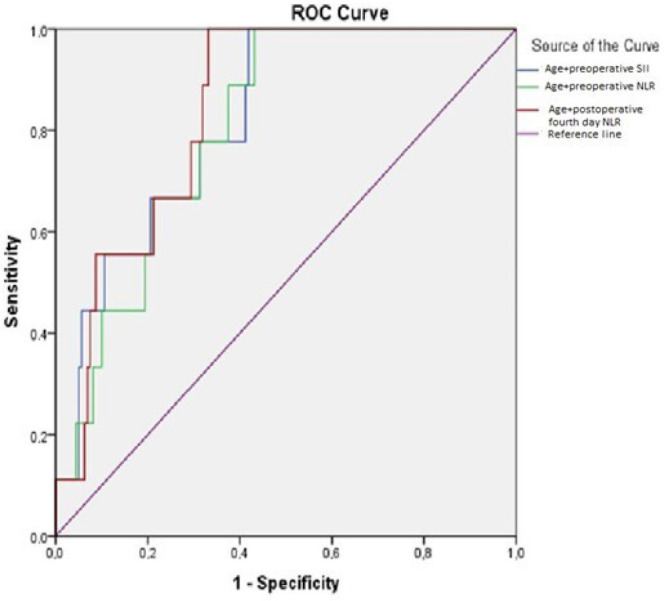
Receiver operating characteristic (ROC) curve for combined parameters. NLR=neutrophil/lymphocyte ratio; SII=systemic immune-inflammation index.

The parameters observed to affect mortality and their combination values underwent ROC analysis. The investigation found that age + preoperative SII, age + preoperative NLR, and age + postoperative fourth day NLR combinations had significant predictive values for detection of 30-day mortality (Table 8, Figure 4).

**Table 8:** Receiver operating characteristic analysis of the combination of some parameters affecting mortality and diagnostic values of the combined parameters for 30-day mortality prediction.

Combination	AUC (95% CI)	Cut-off†	*P*-value	Sensitivity (%)	Specificity (%)
Age + preoperative SII (× 10^9^/L)	0.802 (0.699-0.906)	0.082	< 0.001	75.0	68.1
Age + preoperative NLR (× 109/L)	0.794 (0.691-0.898)	0.087	< 0.001	75.0	68.8
Age + postoperative 4^th^ day NLR (× 10^9^/L)	0.839 (0.749-0.928)	0.049	0.001	66.7	70.6

Reference category: survival group

†Predictive values obtained from the logistic regression model: Ln (p[x]/1-p[x]) =constant+B₁X₁ +B₂X₂ (B=LR coefficient, x=parameter, constant=LR constant coefficient)

AUC=area under curve; CI=confidence interval; NLR=neutrophil/lymphocyte ratio; SII=systemic immune-inflammation index

## DISCUSSION

After trauma is experienced by tissue, the rapid but controlled response of the immunological system forming at cellular or humoral level for tissue-protective purposes is called inflammation^[10^,^11]^. As with all types of surgery, there are many factors that trigger the inflammation response in cardiac surgery; multiple incisions, traction-linked trauma, CPB duration, hypothermia, blood and blood products, and use of foreign material^[12]^. Thrombocytosis and leukocytosis may be encountered during the inflammatory response. In recent years, rather than single numerical values for these parameters, their ratios of NLR, PLR, and SII have become more valuable and provide ideas about disease progression, complications that may develop, prognosis, and mortality in several pathological situations.

As our study included a clear proportion of patients with rheumatic heart valve disease diagnosis (58%) and this situation may affect SII values, patients were divided into two diagnostic groups of rheumatological and degenerative disease and statistical investigations were performed. As a result of the analysis, there were no significant differences for the preoperative, postoperative first day, and postoperative fourth day SII values between the rheumatological and degenerative groups (*P* > 0.1). Additionally, the SII values were lowest in the preoperative period and highest on the postoperative first day among the examined parameters (age, sex, valve, and diagnostic groups), and this was significant (*P* < 0.001).

Two different studies researching the effect of SII on prognosis (one investigated off-pump coronary artery bypass grafting patients, the other investigated chronic heart failure patients) similarly found that SII predicted poor outcomes^[13^,^14]^. Another study, including aorta and mitral valve surgery cases and calculating preoperative SII, divided patients into two groups according to prognosis. The SII levels for patients in the poor outcome group were significantly higher than for patients in the positive outcome group (658.40 ± 436.29 *vs.* 335.72 ± 174.76; *P* < 0.001). The high SII group had significantly higher poor outcome incidence (*P* < 0.001) and 30-day mortality (*P* < 0.001) rates^[15]^. A study including patients with transcatheter aorta valve replacement researched the effect of NLR and PLR on 30-day outcomes. In conclusion, high NLR and PLR values were associated with increased 30-day negative outcomes^[16]^.

In our study, the preoperative CRP (*P* = 0.003), NLR (*P* = 0.003), and SII (*P* = 0.02) values, postoperative fourth day NLR value (*P* < 0.001), and age (*P* < 0.001) were observed to be significantly higher in the mortality group. Additionally, SII values at the three different time points were analyzed with ROC for30-day mortality prediction. The investigation observed that preoperative SII values had predictive features (592.11 × 109/L cut-off value, sensitivity 81.3%, and specificity 52.5%). Again, the combined values of some parameters observed to affect mortality in our study were analyzed with ROC for prediction of 30-day mortality. Age + preoperative SII (sensitivity 75%, specificity 68.1%), age + preoperative NLR (sensitivity 75%, specificity 68.8%), and age + postoperative fourth day NLR (sensitivity 66.7%, specificity 70.6%) combinations were identified to be significant predictive factors for detection of 30-day mortality.

A study of isolated tricuspid valve surgery found that the group with high preoperative SII values had higher 30-day major complication rates compared to the group with low SII (*P* = 0.001). Additionally, PLR, NLR, and SII were reported to be prognostic factors for 30-day major complications in the study. The same study observed significant differences in hospitalization duration (28.2 ± 27.6 days - 13.3 ± 18.2 days, *P* = 0.001)^[17]^. Another study found that the patient group with high SII had significantly high readmission to hospital rates within 30 days compared to the low SII group (*P* = 0.026)^[15]^. In our study, the presence of correlations between preoperative SII, postoperative first day SII, and postoperative fourth day SII parameters with ICU admission duration was investigated. Only postoperative fourth day SII values were identified to have a statistically significant, non-linear positive correlation with ICU admission duration (*P* < 0.001, *Ρ* = 0.308).

For patients with MVR, 30-day mortality varies between 4.9% and 9%^[18^,^19]^. A single-center study observed numerically higher survival rates in the early postoperative period for patients using biological valves, while this rate changed in favor of mechanical valves after three years^[18]^. A study comparing long-term survival (15 years) did not find a significant statistical difference in terms of survival between mechanical and biological MVR^[20]^. Another study including patients over 65 years with MVR reported death rates of 5.6% with mechanical valve and 8% with biological valve^[21]^. In our study, the 30-day mortality rates were 9%, similar to the literature. The mortality rates were 3.9% in rheumatic heart valve disease group and 16.2% in the degenerative group (*P* = 0.005). While the mortality rate was 28% for the bioprosthetic valve group, it was 6% in the mechanical valve group (*P* = 0.002). The mean age was 52.7 years in the mechanical valve group and 72.0 years in the biological group. We believe this result is associated with the bioprosthetic valve group and degenerative diagnosis group comprising patients with advanced age and high comorbidities.

### Limitations

Limitations of the study include its retrospective character, inclusion of patients operated only with sternotomy, not including patients operated with thoracotomy, and only showing correlations of SII with 30-day early-term prognosis and mortality. In addition, separate analyses were not performed for each type of heart valve due to insufficient numbers. A roller pump was used in our study. The lack of any data regarding the use of centrifugal pumps can be considered as another limitation.

## CONCLUSION

In conclusion, when investigated in terms of 30-day mortality prediction, significant positive correlations were observed between age, preoperative SII, preoperative NLR, and postoperative fourth day SII and NLR values with mortality. The combinations of age + preoperative SII, age + preoperative NLR, and age + postoperative fourth day NLR were found to have significant predictive effect for 30-day mortality. Despite all these significant results, the clinical applicability of SII may be poor.

### Ethical Approval

Permission was granted by the local ethics committee for the study protocol (2022/3653), and every patient provided written informed consent.
